# Relationships between Molecular Characteristics of Novel Organic Selenium Compounds and the Formation of Sulfur Compounds in Selenium Biofortified Kale Sprouts

**DOI:** 10.3390/molecules28052062

**Published:** 2023-02-22

**Authors:** Paweł Zagrodzki, Agnieszka Wiesner, Monika Marcinkowska, Marek Jamrozik, Enrique Domínguez-Álvarez, Katarzyna Bierła, Ryszard Łobiński, Joanna Szpunar, Jadwiga Handzlik, Agnieszka Galanty, Shela Gorinstein, Paweł Paśko

**Affiliations:** 1 Department of Food Chemistry and Nutrition, Faculty of Pharmacy, Medical College, Jagiellonian University, Medyczna 9, 30-688 Kraków, Poland; 2 Department of Medicinal Chemistry, Faculty of Pharmacy, Jagiellonian University Medical College, 9 Medyczna Str., 30-688 Cracow, Poland; 3 Instituto de Química Orgánica General (IQOG), CSIC, Juan de la Cierva 3, 28006 Madrid, Spain; 4 IPREM—Institute of Analytical and Physical Chemistry for the Environment and Materials, CNRS-UPPA UMR 5254, Hélioparc, 64053 Pau, France; 5 Department of Chemistry, Warsaw University of Technology, ul. Noakowskiego 3, 00-664 Warsaw, Poland; 6 Department of Technology and Biotechnology of Drugs, Faculty of Pharmacy, Medical College, Jagiellonian University, Medyczna 9, 30-688 Kraków, Poland; 7 Department of Pharmacognosy, Faculty of Pharmacy, Medical College, Jagiellonian University Medyczna 9, 30-688 Kraków, Poland; 8 Institute for Drug Research, School of Pharmacy, Faculty of Medicine, The Hebrew University of Jerusalem, Jerusalem 9112001, Israel

**Keywords:** selenium, sulfur compounds, biofortification, brassica sprouts, chemometric analysis

## Abstract

Due to problems with selenium deficiency in humans, the search for new organic molecules containing this element in plant biofortification process is highly required. Selenium organic esters evaluated in this study (E-NS-4, E-NS-17, E-NS-71, EDA-11, and EDA-117) are based mostly on benzoselenoate scaffolds, with some additional halogen atoms and various functional groups in the aliphatic side chain of different length, while one compound contains a phenylpiperazine moiety (WA-4b). In our previous study, the biofortification of kale sprouts with organoselenium compounds (at the concentrations of 15 mg/L in the culture fluid) strongly enhanced the synthesis of glucosinolates and isothiocyanates. Thus, the study aimed to discover the relationships between molecular characteristics of the organoselenium compounds used and the amount of sulfur phytochemicals in kale sprouts. The statistical partial least square model with eigenvalues equaled 3.98 and 1.03 for the first and second latent components, respectively, which explained 83.5% of variance in the predictive parameters, and 78.6% of response parameter variance was applied to reveal the existence of the correlation structure between molecular descriptors of selenium compounds as predictive parameters and biochemical features of studied sprouts as response parameters (correlation coefficients for parameters in PLS model in the range—0.521 ÷ 1.000). This study supported the conclusion that future biofortifiers composed of organic compounds should simultaneously contain nitryl groups, which may facilitate the production of plant-based sulfur compounds, as well as organoselenium moieties, which may influence the production of low molecular weight selenium metabolites. In the case of the new chemical compounds, environmental aspects should also be evaluated.

## 1. Introduction

Selenium (Se) is an essential element for humans, the deficiency of which can cause clinical disorders, such as Kashin-Beck and Keshan diseases [1]. Biofortification of plants with Se is one of the strategies of searching for novel candidates for functional foods, which can serve as Se sources in the diet and at least partially solve the problem of its deficiency. Although selenium is not an essential element for plants, it plays an important role in their growth when used in proper doses. However, due to the differences in Se accumulation among plants, and the risk of harmful impact, particular care should be taken in the case of plant biofortification with this element. Thus, selenium tolerance for different plant species and varieties should be assessed each time in a preliminary experiment.

Actually, our previous studies on the toxic effects of selenium on plants indicated some limitations in this respect (e.g., high doses of selenium adversely affected seeds germination), especially when using inorganic selenium compounds [2,3]. Such forms have been so far most widely used in the plant biofortification process because they are easily absorbed and metabolized in the tissue and converted into organic forms, e.g., selenomethionine and selenocysteine [4].

It is well known that organic forms of selenium are more bioavailable for animals and humans [4]. Therefore, the development and introduction of new organic molecules containing selenium moieties, which, when used for plants biofortification, could provide a food product with increased bioavailability of the compounds, is highly expected.

To meet these expectations, in our previous research we synthesized novel organic selenium derivatives (and described the effect of enriching kale sprouts with the compounds on both phytochemical profile and biological activity of these plants [5,6]. The compounds belong mostly to the class of organic esters (E-NS-4, E-NS-17, E-NS-71, EDA-11, EDA-117) and contain an aromatic ring. Some of them were enriched with additional nitrogen or halogen atoms, as well as various functional groups in the aliphatic side chain of different length. Thus, these compounds enclose benzoselenoate scaffolds. One compound (WA-4b) has a distinctly different structure, as it possesses a phenylpiperazine moiety. Biofortification of kale sprouts with the compounds caused remarkably high increase in the content of the analyzed sulfur compounds, especially some isothiocyanates (ITC) in comparison to the untreated sprouts, most evident in the case of phenylselenyl derivative (WA-4b), which strongly stimulated the synthesis of benzyl-, butyl-, phenyl-, and phenethyl- isothiocyanate in kale sprouts [6]. Moreover, sulforaphane content increased more than twofold in WA-4b biofortified sprouts. Similar impact on sulforaphane amount was noted in the case of the sprouts biofortified with other selenium compounds used in the study, such as EDAG-11, EDA-117, E-NS-17, and EDA-71, which also significantly affected the production of allyl isothiocyanate [6].

Furthermore, most of the novel selenium compounds also significantly increased the amounts of assessed glucosinolates (GLS) and phenolic acids (mainly chlorogenic, protocatechuic, and sinapic) in the sprouts. We also evaluated the impact of the tested selenium compounds on the antioxidant and anti-inflammatory properties, as well as the cytotoxic activity of sprouts on normal and cancer cells of the gastrointestinal tract, prostate, and thyroid [6]. It is worth noting that the Se-biofortified kale sprouts were non-toxic for normal human cells (PNT2 and Nthy-ori 3-1) used in the study, in wide range of the tested concentrations (0.05–0.5 mg/mL) [6]. In addition, the compounds tested have undergone selectivity assays [7] and ADMETox evaluations [8], which showed that they are selective towards cancer cells (therefore, less toxic to non-tumor cells) and non-mutagenic [7,8].

We presented detailed characteristics of all the above-mentioned Se compounds in our former work, pointing to certain differences in their physicochemical properties [5]. We found that less than 10% of supplemented selenium was incorporated into the kale sprouts as selenomethionine, while at least a dozen other low-molecular-weight organic metabolites of primary selenium compounds were synthesized in the sprouts during their enrichment. Moreover, we did not find any selenium compounds with selenol motifs, but we confirmed the presence of several compounds possessing the Se–S bonds [5].

All these observations prompted us to undertake a deeper insight into the relationships between Se biofortification of kale sprouts and the content of bioactive sulfur compounds in the search for the derivative with optimal effect. We hypothesized that some structural elements in the examined Se derivatives can influence the synthesis of sulfur compounds in kale sprouts with different potency. To verify the hypothesis, we gathered molecular characteristics of primary selenium compounds and used statistical partial least square (PLS) analysis to check the existence of the correlation structure between molecular descriptors of selenium compounds as predictive parameters and chemical or biochemical features of studied sprouts as response parameters. Molecular characteristics can be approached using constitutional, topological, hydrogen bonding, and charge-based molecular descriptors, as they all are mathematical representations of the physical and chemical molecules’ properties [9,10]. We also established that such a strategy could enable us to preselect the structural elements important for further development of Se derivatives with optimal enhancement effect on the sprouts.

## 2. Results and Discussion

PLS is one of the methods for analyzing multivariate data that can be divided into sets of predictor and response parameters. It is implemented in such a way that the block of the original predictor variables is spanned by mutually orthogonal latent variables, which are linear combinations of the predictor variables. The response variables can be modelled in the same way. The latent variables can be found by an iterative procedure to provide maximal fit to the path model, i.e., to combine the predictor and response parameters with common latent components, and show—in such way—the joint correlation structure between them. The parameters with large absolute values of their coordinates (>0.3) on the first two latent components in the PLS model are assumed to determine the axes (latent components) of the new coordinate system (in PLS model) to the greatest extent. To express the strength of bivariate associations between such parameters, the cosines of the corresponding angles (i.e., correlation coefficients) were calculated. The “corresponding angle” means the angle determined by the two lines connecting the origin with coordinates of both parameters on the PLS loadings plot. Although such modelling is associated with reduction of original data dimensionality, a large amount of information from the original data is still retained. The mathematical details of the PLS method are described elsewhere [10,11].

In the role of response parameters in our PLS model, we first tested the following groups of parameters: (i) indices of plant selenium status (described as the concentration of total selenium, selenomethionine (SeMet), Se(IV) and Se-methylselenocysteine (SeMetSeCys) in sprouts and in the water and proteolytic extracts); (ii) phenolic acids (chlorogenic, isochlorogenic, protocatechuic, caffeic, and sinapic acids) content and the indices of antioxidant status (FRAP, DPPH); (iii) sulfur compounds (allyl isothiocyanate, butyl isothiocyanate, diindolylmethane, glucoerucin, glucoiberin, indol-3-carbinol, phenyl isothiocyanate, phenethyl isothiocyanate, progoitrin, sinigrin, and sulforaphane) concentrations; (iv) indices of inflammation process, i.e., cytokines (TNF-alpha and IL-6) and nitric oxide; (v) indices of chemopreventive potential, i.e., data of cytotoxic activity obtained from the work with human cancer and normal cells (DU-145, PC-3, PNT2, Caco-2, HT-29, HepG2, FTC-133, 8505C, and Nthy-ori 3-1) [5,6].

Finally, we revealed PLS model fulfilling cross-validation criteria only for sulfur compounds in Se-biofortified kale sprouts as response parameters. Therefore, in the present study, we investigated possible correlations between the chemical structure of organoselenium biofortifiers and the level of sulfur compounds produced by kale sprouts.

In particular, the PLS model correlated the predictive parameters (here: MlogP (Moriguchi octanol-water partition coefficient), MolVol (liquid molar volume), N_Atoms (number of atoms), N_Carbon (number of carbon atoms), N_Hydrgn (number of hydrogen atoms), N_Oxygen (number of oxygen atoms), N_Bonds (number of bonds), F_SgleB (single bonds as fraction of total bonds), F_DbleB (double bonds as fraction of total bonds), F_TpleB (triple bonds as fraction of total bonds), Carbonyl_C=O (number of carbonyl (ketone or aldehyde) groups), HBAo (number of oxygen-based hydrogen bond acceptors), HBAch (the sum of estimated NPA partial atomic charges on hydrogen bond acceptors), BAoch (the sum of estimated NPA partial atomic charges on oxygen-based hydrogen bond acceptors), HBAnch (sum of estimated NPA partial atomic charges on nitrogen-based HB Acceptors)) and the response ones, which were defined in our study as the content of: allyl isothiocyanate, butyl isothiocyanate, glucoerucin, glucoiberin, indol-3-carbinol, phenyl isothiocyanate, phenethyl isothiocyanate, progoitrin, and sinigrin. Several other parameters, originally included in the analysis (MWt (molecular weight), N_Ntrgen (number of nitrogen atoms), F_AromB (aromatic bonds as fraction of total bonds), Nitrile C#N (number of nitrile groups), T_PSA (topological polar surface area), and HBAn (number of nitrogen-based hydrogen bond acceptors)) were finally discarded from the model, as they were considered non-informative, i.e., they had relatively small loadings on both latent components.

**Figure 1:**
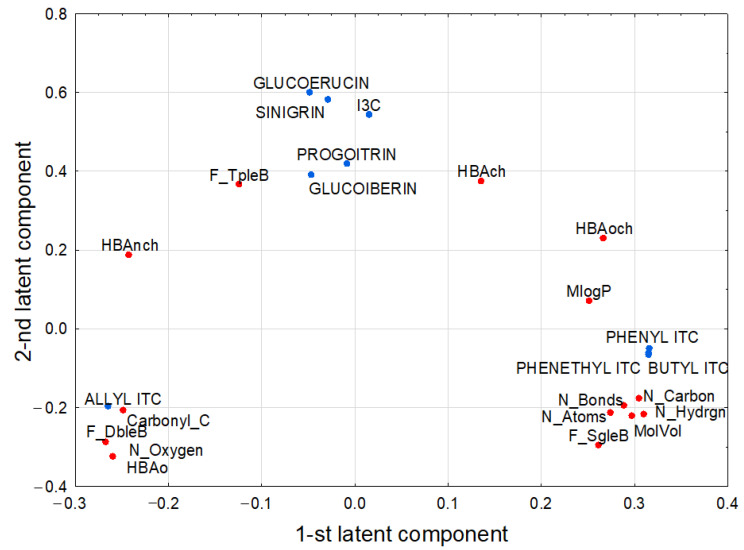
Loadings of first and second latent components in the PLS model. The model correlated the predictive parameters (MlogP, MolVol, N_Atoms, N_Carbon, N_Hydrgn, N_Oxygen, N_Bonds, F_SgleB, F_DbleB, F_TpleB, Carbonyl_C=O, HBAo, HBAch, BAoch, HBAnch) and response ones (allyl isothiocyanate (ALLYL ITC), butyl isothiocyanate (BUTYL ITC), glucoerucin, glucoiberin, indol-3-carbinol (I3C), phenyl isothiocyanate (PHENYL ITC), phenethyl isothiocyanate (PHENYLETHYL ITC), progoitrin, and sinigrin).

The model had two significant components with eigenvalues higher than 1 (equaled 3.98 and 1.03, respectively) and explained 83.5% of variance in the predictive parameters and 78.6% of response parameter variance. The loadings for the first two latent components are shown on Figure 1.

The first latent component in this model had positive weights predominantly for N_Carbon, N_Hydrgn, and response ones: phenyl isothiocyanate, phenethyl isothiocyanate, and butyl isothiocyanate, all being strongly positively correlated (Table 1).

**Figure 2:**
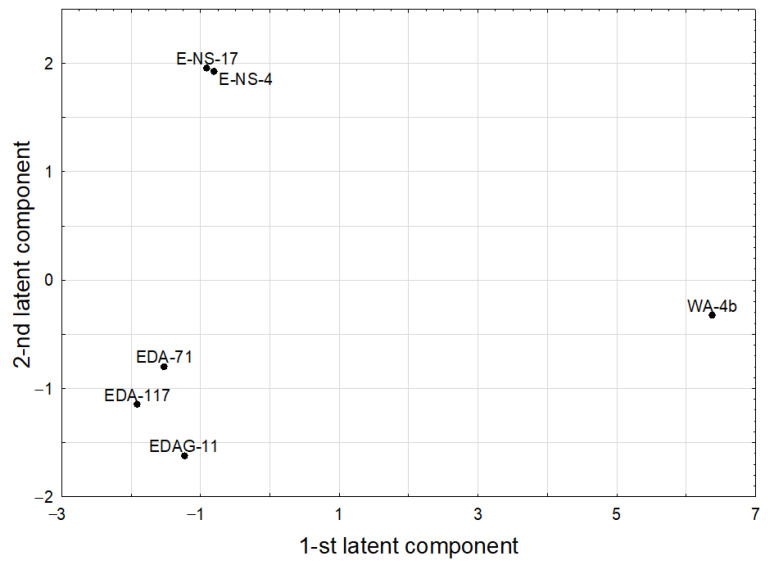
Score scatterplot of PLS model.

The second latent component was loaded positively mainly by the response parameters: glucoerucin, sinigrin, indol-3-carbinol, progoitrin, glucoiberin, and two predictive ones, F_TpleB and HBAch, while it was negatively loaded mainly by HBAo and N_Oxygen. The last two were strongly negatively correlated with the former ones. The score scatterplot of PLS model, whose points of original selenium compounds in the space determined by first two latent components were shown on Figure 2.

### 2.1. Relationships between Molecular Descriptors

In the analyzed organoselenium compounds, all oxygen atoms were also hydrogen bond acceptors, hence, HBAo = N_Oxygen, with the respective correlation coefficient equaling 1. There was a strong positive correlation between the number of carbon and hydrogen atoms. In the analyzed organoselenium compounds, hydrogen atoms were predominantly combined with carbon atoms (exception: -NH_2_ group in compound EDA-117), so the number of hydrogen atoms increased along with the number of carbon atoms, in direct proportion. On the other hand, a negative correlation was found between the number of oxygen atoms and the fraction of triple bonds in all bonds in the molecule. Oxygen atoms form either single or double bonds. Therefore, the greater number of oxygen atoms implies a smaller fraction of triple bonds in the molecule. A negative correlation was also observed between the fraction of triple bonds in all bonds in a molecule and the number of oxygen-based hydrogen bond acceptors, according to the rule: the more triple bonds a molecule contains, the less likely the oxygen-based hydrogen bond acceptors are. There was a negative correlation between the number of oxygen atoms/number of oxygen-based hydrogen bond acceptors and the sum of the estimated partial charges on the hydrogen bond acceptors. Since these are negative charges, the number indicated by the descriptor decreases with the increasing charge as it becomes more negative. Hence, more oxygen atoms in a molecule result in higher absolute sum of the estimated partial charges on hydrogen bond acceptors.

### 2.2. Relationships between Sulfur Compounds

Strong positive correlations were found between the content of the three isothiocyanates, namely, phenethyl ITC, butyl ITC, and phenyl ITC, which are formed through the hydrolysis of glucosinolates. Strong positive correlations were also observed between the content of glucoiberin, glucoerucin, sinigrin, and progoitrin, the glucosinolates that are derivatives of the same amino acid—methionine. Moreover, the mentioned glucosinolates were positively correlated with the content of indole-3-carbinol, a breakdown product of these compounds.

### 2.3. Relationships between Descriptors (F_TpleB, HBAch) and Sulfur Compounds

The fraction of triple bonds in all bonds in the molecule (F_TpleB) strongly positively correlated with the content of the sulfur compounds: glucoiberin, glucoerucin, sinigrin, I3C, and progoitrin. In the analyzed organoselenium compounds, the triple bond was present in the nitrile group only (-CN). Thus, the presence of a nitrile group in the organoselenium compound was associated with higher content of the mentioned sulfur compounds in the biofortified sprouts. This could be explained by the fact that the nitriles are one of the possible hydrolysis products of glucosinolates, so a compound with a nitrile group could potentially reverse the hydrolysis process (through the negative feedback loop) or compete for the nitrile specifier protein (NSP) and thus decrease the hydrolysis of sulfur compounds. There was also a strong positive correlation between the sum of the estimated partial charges on hydrogen bond acceptors and the synthesis of sulfur compounds: glucoiberin, glucoerucin, sinigrin, I3C, and progoitrin, conforming to the rule that the higher the number indicated by the descriptor is, the higher is the content of the sulfur compounds. However, it must be taken into account that the charge on the hydrogen bond acceptors is negative. Thus, the more acceptors are present, the more negative the sum of partial charges is, and so is the smaller the number indicated by the descriptor. So, the absolute value of the sum of the partial charges should be taken into account. However, our results indicate that higher absolute sum of the partial charges on the hydrogen bond acceptors did not favor the synthesis of the sulfur compounds. Hydrogen bond acceptors are highly electronegative atoms, represented in the analyzed organoselenium compounds by oxygen (correlation described below), nitrogen, and chlorine atoms, which tend to donate electrons or accept hydrogens due to their oxidizing potential.

### 2.4. Relationships between Descriptors N_Carbon and N_Hydrgn and Sulfur Compounds

The numbers of carbon and hydrogen atoms in the analyzed organoselenium compounds exhibit a strong positive correlation with the content of isothiocyanates—phenethyl ITC, butyl ITC, and phenyl ITC. Obviously, large molecules have a higher number of carbon and hydrogen atoms. However, among the compounds of similar size, a higher number of carbon and hydrogen atoms suggests lower polarity of the compound, associated with the presence of aromatic rings/long aliphatic chains. Among the analyzed organoselenium compounds, the least polar compound, WA-4b, had the highest molar mass and the number of carbon and hydrogen atoms. The other compounds were characterized by lower molar masses and lower numbers of carbon and hydrogen atoms, while they had more electronegative atoms, thus being more polar.

### 2.5. Relationships between Descriptors (N_Oxygen, HBAo) and Sulfur Compounds

The number of oxygen atoms/number of oxygen-based hydrogen bond acceptors (N_Oxygen/HBAo) correlated negatively with the content of the sulfur compounds—glucoiberin, glucoerucin, sinigrin, I3C, and progoitrin. In the analyzed compounds, oxygen was present in the form of a carbonyl group (>C=O), the presence of which, however, did not favor the synthesis of the above-mentioned sulfur compounds. A possible explanation for the observed correlation, and also the one between the sum of the estimated partial charges on hydrogen bond acceptors and the synthesis of the sulfur compounds, is that several enzymes involved in the synthesis of glucosinolates: 3-isopropyl malate dehydrogenase (IPMDH), APS kinase, and FRY1, may be more active when present in their reduced form [12]. Meanwhile, the presence of the compounds containing a lot of oxygen atoms/oxygen-based hydrogen bond acceptors favors the oxidation rather than the reduction processes.

### 2.6. Other Implications of PLS Model

The structures of the analyzed Se derivatives E-NS-4 and E-NS-17 differ in only one substituent, which in both cases is a halogen—strongly electronegative atoms, Cl and F, respectively. Thus, a similar effect of both molecules on the synthesis of sulfur compounds is to be expected. Compound WA-4b differs in its chemical structure from all the others, as it lacks electronegative oxygen atoms and halogens. It also does not have a nitrile group. It has significantly more carbon and hydrogen atoms than other analyzed Se derivatives, and it is also characterized by higher molar mass and lower polarity.

**Figure 3:**
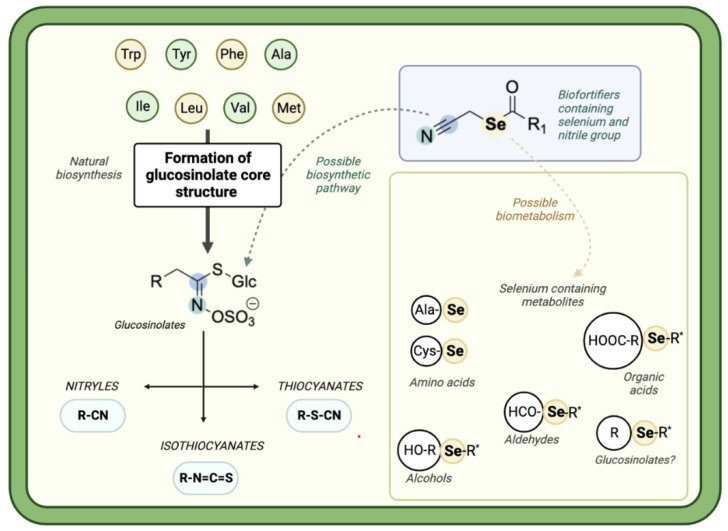
Possible implications of organic selenium compounds biofortification on the synthesis of active compounds in kale sprouts. * Selenosulfides, Selenoethers.

Interestingly, we found no correlation between the number of selenium atoms and the final amount of the sulfur compounds. This may suggest that the plant utilizes selenium atoms incorporated inside the fortifiers directly in the biochemical pathway, leading to the formation of low molecular weight selenium metabolites, rather than in the synthesis of sulfur bioactive compounds. The present results strongly suggest that it is the nitryl group of the fortifiers that may have a direct influence on the plant’s synthesis of sulfur compounds (Figure 3), serving as the nitrogen source.

This figure was prepared on the basis of information drawn from plant physiology studies [12,13,14,15,16] and with implementation of newly obtained chemometric results.

### 2.7. General Remarks

It is known that sulfur-containing precursors have a large impact on glucosinolate accumulation in plant tissue [12]. The possible ways of regulating plants’ secondary metabolism by their biofortification with different compounds has been described as the changes occurring in primary sulfur/nitrogen metabolism, hormonal regulation, redox metabolism, as well as at the transcriptomic level of secondary metabolite biosynthesis. However, glucosinolates are regulated not only by sulfate nutrition, but also by other nutrients, such as nitrogen and selenium, which can affect both glucosinolate synthesis and accumulation.

It is a very interesting observation, partially in line with the concept by Skrypnik et al., [17], that selenium accumulation influences gene expression, similar to the state of sulfur deficiency. The presence of Se affects the expression of key genes encoding sulfur transporters and the enzymes that regulate sulfur metabolism. The absorption and assimilation of sulfur by plants are coordinated with the absorption and assimilation of nitrogen, and selenium also affects nitrogen metabolism [17]. The change in nitrogen assimilation has serious consequences for the synthesis of all nitrogen-containing metabolites, including amino acids and their derivatives glucosinolates.

It is known that inorganic nitrogen compounds may have an influence on the synthesis of sulfur compounds in edible Brassica plants. Zhao et al. [18] found that ammonium nitrate biofortification in rape seeds resulted in the abundance of four alkenyl glucosinolates. The increase in the nitrogen rate boosted the proportion of progoitrin (2-hydroxybut-3-enyl) at the loss of glucobrassicanapin (pent-4-enyl), and to a lesser extent gluconapoleiferin (2-hydroxypent-4-enyl), in the low erucic acid and low glucosinolates rape variety, but at the expense of gluconapin (but-3-enyl) in low erucic acid rape variety. Additionally, it was shown that high nitrogen biofortification promoted the hydroxylation step from but-3-enyl to 2-hydroxybut-3-enyl glucosinolates. Kopsell et al. [19] noticed that adding calcium nitrate tetrahydrate and potassium nitrate to watercress increased glucobrassicin, 4-methoxyglucobrassicin, and gluconasturriin levels. Omirou et al. [20] evaluated the influence of nitrogen, added as ammonium nitrate and magnesium nitrate, on the GLS concentration in broccoli, and they described the positive response of the plants to the nitrogen delivered, which had a higher impact on indoles compared to aliphatic GSL content. De Maria et al. [21] evaluated the biofortification effect of ammonium nitrate and ammonium sulphate on the content and composition of nine GLSs in horseradish. Total GLS amount showed the greatest values at the beginning of plant regrowth and then decreased throughout the plant development until the end of the growing period. This is also in agreement with our results for the sprouts treated with the organoselenium compounds rich in different nitrogen elements, with the observed extremely high amount of GLS. The GLS classes varied in different ways, depending on the developmental stage and fertilization, showing the highest percentage increase at the beginning of plant regrowth: aliphatic GLS (glucoiberin, sinigrin, and gluconapin) increased by 150%, while aromatics (glucobarbarin, epiglucobarbarin, and gluconasturriin) and indoles (4-methoxyglucobrassicin, glucobrassicin) increased up to 35% with nitrogen biofortification, respectively. The results suggest that the fertilization led to the modulation of GLS content and their composition in plants [21].

GSL content in the plants may be not significantly affected by Se fertilization [22], which was also confirmed by our study. One exception was noted for indol-3-ylmethyl glucosinolate, which increased by Se treatment in radish. The transcript of the genes involved in aliphatic GSL synthesis declined with Se treatment, while that of the genes involved in indole GSL synthesis had a tendency to increase. Adenosine 5′-phosphosulfate kinase (APS kinase) transcript abundance increased significantly following Se treatment. McKenzie et al. [22] indicated that the increased APS kinase expression in response to Se treatment is the part of a general protection mechanism controlling the uptake of sulfur and the production of GSL.

Other studies [17,23] have shown that selenium significantly reduced the expression of genes: BCAT4 and MAM1 involved in chain elongation; CYP79B2, CYP79F1, CYP83B1, and CYP83A1, involved in the formation of the core glucosinolates structure; and UGT74B1 and FMO2, involved in the secondary modifications of these compounds in broccoli. In addition, the exposure to selenate suppressed the genes encoding the transcription factors MYB28 and MYB34, which regulate aliphatic- and indole-GSL synthesis.

We are aware that selenium added during the biofortification process may be incorporated not only into selenoproteins or selenoaminoacids, but also into other structures, such as selenium-containing glucosinolates (Se-GSL) [24]. It was proved that the level of various Se–GSL significantly increased during plant development under selenium biofortification. Brassica crops supplied with selenate were able to form Se-GSL, with a methylselenoalkyl group that was likely derived from selenomethionine and Se-GSL accounted for 60% of the concentrations of their sulfur analogs [25]. The production of Se–GLS following Se-fertilization has implications for human health, as the synthetic Se-containing isothiocyanates are reported to be more potent cytotoxic compounds than their S counterparts [26].

## 3. Materials and Methods

### 3.1. Biofortification Process and Extract Preparation

**Figure 4:**
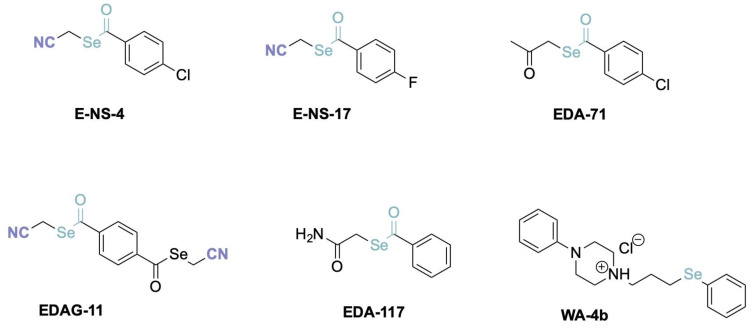
Chemical structures of selenium compounds used in the kale sprouts biofortification.

The biofortification process was previously described in detail [5,6]. Briefly, kale (*Brassica oleracea* L. var. *sabellica)* seeds were immersed for 3 h in milliQ water without (control) or with the organic selenium compounds, denoted as E-NS-4, E-NS-17, EDA-71, EDAG-11, EDA-117, or Wa-4b (selenium concentrations of 15 mg/L). Detailed chemical structures of used selenium compounds were presented in Figure 4. Then, the seeds were transferred to a plastic sprouts’ maker and grown for seven days after seeding in the greenhouse of Garden of the Medicinal Plants, Faculty of Pharmacy Medical College, at 25 ± 2 °C, 70% of humidity, in sunlight exposure (10 h/day), being watered every day with 150 mL freshly prepared selenium solutions.

For sulfur compounds analysis, the fresh sprouts were mashed and then incubated in a closed forced-air oven for 4 h to promote hydrolysis of GLS to ITC by myrosinase [6]. The obtained material was then extracted with methanol in a Soxhlet apparatus for 3 h.

### 3.2. Identification of Sulfur and Selenium Compounds in Kale Sprouts after Biofortification Process

Quantitative and semi-quantitative UPLC-MS/MS analysis of GLS, ITC, and indole isothiocyanates was applied with using UPLC-MS/MS system consisted of a Waters ACQUITY^®^ UPLC^®^ (Waters Corporation, Milford, MA, USA) coupled to a Waters TQD mass spectrometer (electrospray ionization mode ESI-tandem quadrupole). Chromatographic separations were carried out using the Acquity UPLC BEH (bridged ethyl hybrid) C18 column, 2.1 × 100 mm, and 1.7 µm particle size. The total selenium content in the sprouts, water extracts, and proteolytic digests was determined by means of ICP-MS (Agilent 7700x, Agilent, Tokyo, Japan). All these procedures were described in [5,6].

### 3.3. Input Data of Molecular Characteristics and Sulfur Compounds

The set of molecular descriptors was computed for each novel selenium compound (Figure 4) using the ADMET Predictor Software (Simulations Plus, Inc., Lancaster, CA, USA) [27]. Due to the fact that the ADMET program was not developed on the basis of such atoms, such as selenium, some parameters were marked by the program as not entirely reliable and, therefore, were removed from further calculations. Several parameters that took text values (and these values were the same for all compounds) were also removed. Of the molecular descriptors those that had a value of zero or showed very little variability for all compounds (i.e., they had only one or two values for all tested compounds) were eventually rejected as well.

The data concerning sulfur compounds in Se-biofortified kale sprouts were taken from our previous papers, which also described sample preparation, extraction procedures, and analytical techniques [5,6].

### 3.4. Chemometric Analysis

The PLS model, which fulfilled cross-validation criteria, was constructed using SIMCA-P v.9 software (Umetrics, Umeå, Sweden). STATISTICA v. 13.3. package (TIBCO Software Inc., Palo Alto, CA, USA) was used for graphic representation of PLS model.

## 4. Conclusions

**Figure 5:**
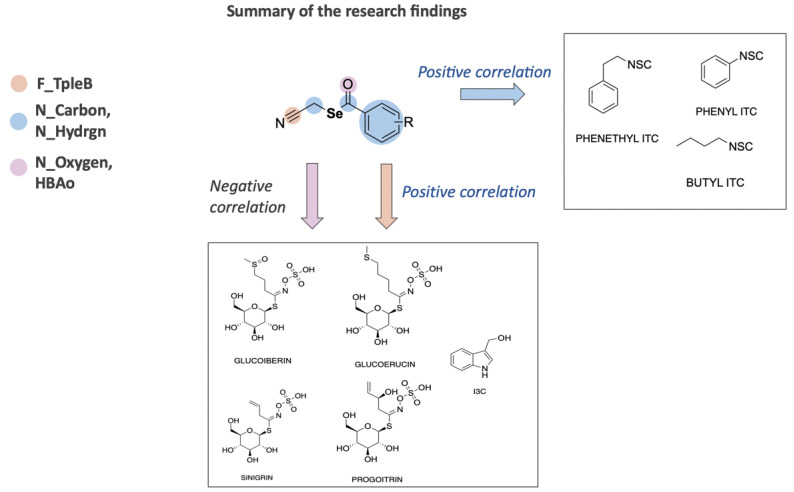
Summary of the main relationship regarding structure of organic selenium compounds and synthesis of different sulfur compounds.

Based on the conducted research, it can be recommended that, when composing the effective biofortifiers in the future, the simultaneous combination of nitryl groups and organoselenium moieties should be considered. The nitryl groups may facilitate the production of plant-based sulfur compounds, similarly to organoselenium moieties, which may additionally influence the production of low molecular weight selenium metabolites. The combination of these two elements in the chemical structures of supplemented compounds would probably be the optimal solution (Figure 5).

It would be also interesting to verify the influence of a biofortifier containing solely nitryl groups on the production of sulfur compounds by plant. Especially, in future work, the Se-GLS and/or GLS content must be monitored using in vitro studies before edible plants or their waist are used for human (functional foods) or animal consumption in order to avoid potential harmful effects.

Considering that current chemical repertoire of potential biofortifiers containing nitryl groups and selenium moieties is limited, the present study can facilitate their design. Compounds bearing nitryl functional group and organoselenium moieties seem to be particularly promising for such purposes, but the aspects of their possible environmental impact should be highlighted and evaluated in the future. This is especially true in the case of nitryl compounds, which can affect soil health, as well as plant productivity [28].

## Figures and Tables

**Table 1:** Correlation coefficients for the pairs of parameters based on first two latent components of the PLS model.

Pairs of Correlated Parameters		Correlation Coefficients	Type of Interaction *
N_Oxygen	HBAo	1.000	1
I3C	PROGOITRIN	1.000	2
PHENETHYL ITC	BUTYL ITC	1.000	2
F_TpleB	HBAch	1.000	2
I3C	SINIGRIN	1.000	2
SINIGRIN	PROGOITRIN	1.000	2
SINIGRIN	GLUCOERUCIN	1.000	2
BUTYL ITC	PHENYL ITC	0.999	2
GLUCOIBERIN	GLUCOERUCIN	0.999	2
PHENETHYL ITC	PHENYL ITC	0.999	2
I3C	GLUCOERUCIN	0.999	2
PROGOITRIN	GLUCOERUCIN	0.998	2
SINIGRIN	GLUCOIBERIN	0.998	2
N_Carbon	N_Hydrgn	0.996	1
I3C	GLUCOIBERIN	0.996	2
PROGOITRIN	GLUCOIBERIN	0.995	2
F_TpleB	GLUCOIBERIN	0.979	3
HBAch	GLUCOIBERIN	0.974	3
F_TpleB	GLUCOERUCIN	0.970	3
HBAch	GLUCOERUCIN	0.965	3
F_TpleB	SINIGRIN	0.962	3
HBAch	SINIGRIN	0.957	3
F_TpleB	I3C	0.956	3
F_TpleB	PROGOITRIN	0.953	3
HBAch	I3C	0.950	3
N_Carbon	PHENETHYL ITC	0.949	4
HBAch	PROGOITRIN	0.947	3
N_Carbon	BUTYL ITC	0.945	4
N_Carbon	PHENYL ITC	0.934	4
N_Hydrgn	PHENETHYL ITC	0.918	5
N_Hydrgn	BUTYL ITC	0.913	5
N_Hydrgn	PHENYL ITC	0.899	5
N_Oxygen	PROGOITRIN	−0.767	6
HBAo	PROGOITRIN	−0.767	6
N_Oxygen	I3C	−0.762	6
HBAo	I3C	−0.762	6
N_Oxygen	SINIGRIN	−0.747	6
HBAo	SINIGRIN	−0.747	6
N_Oxygen	GLUCOERUCIN	−0.727	6
HBAo	GLUCOERUCIN	−0.727	6
N_Oxygen	GLUCOIBERIN	−0.699	6
HBAo	GLUCOIBERIN	−0.699	6
N_Oxygen	F_TpleB	−0.537	1
F_TpleB	HBAo	−0.537	1
N_Oxygen	HBAch	−0.521	1
HBAo	HBAch	−0.521	1

* Type of interaction due to the parameters present in it: 1—dependencies between descriptors; 2—relationships between sulfur compounds; 3—relationships between descriptors (F_TpleB, HBAch) and sulfur compounds; 4—relationships between the descriptor N_Carbon and sulfur compounds; 5—relationships between the descriptor N_Hydrgn and sulfur compounds; 6—relationships between descriptors (N_Oxygen, HBAo) and sulfur compounds.

## Data Availability

On request.

## Abbreviations

ALLYL ITC	allyl isothiocyanate
APS kinase	adenosine 5′-phosphosulfate kinase
BAoch	the sum of estimated NPA partial atomic charges on oxygen-based hydrogen bond acceptors
BUTYL ITC	butyl isothiocyanate
Carbonyl_C=O	number of carbonyl (ketone or aldehyde) groups
F_AromB	aromatic bonds as fraction of total bonds
F_DbleB	double bonds as fraction of total bonds
F_SgleB	single bonds as fraction of total bonds
F_TpleB	triple bonds as fraction of total bonds
GLS	Glucosinolates
HBAch	the sum of estimated NPA partial atomic charges on hydrogen bond acceptors
HBAn	number of nitrogen-based hydrogen bond acceptors
HBAnch	sum of estimated NPA partial atomic charges on nitrogen-based HB Acceptors
HBAo	number of oxygen-based hydrogen bond acceptors
I3C	indol-3-carbinol
IPMDH	3-isopropyl malate dehydrogenase
ITC	Isothiocyanates
MlogP	Moriguchi octanol-water partition coefficient
MolVol	liquid molar volume
MWt	molecular weight
N_Atoms	number of atoms
N_Bonds	number of bonds
N_Carbon	number of carbon atoms
N_Hydrgn	number of hydrogen atoms
N_Ntrgen	number of nitrogen atoms
N_Oxygen	number of oxygen atoms
Nitrile C#N	number of nitrile groups
NPA (Partial Atomic Charge)	Natural population analysis of partial atomic charge
NSP	nitrile specifier protein
PHENYL ITC	phenyl isothiocyanate
PHENYLETHYL ITC	phenethyl isothiocyanate
PLS	partial least square
Se-GSL	selenium-containing glucosinolates
SeMet	Selenomethionine
SeMetSeCys	Se-methylselenocysteine
T_PSA	topological polar surface area
